# Systematic identification and quantification of factors and their interactions with age, sex, and panel wave influencing cognitive function in Korean older adults

**DOI:** 10.3389/fpubh.2025.1547575

**Published:** 2025-02-03

**Authors:** Eunmi Kim, Jinkyung Oh, Jungsoo Gim, Iksoo Huh

**Affiliations:** ^1^College of Nursing, Seoul National University, Seoul, Republic of Korea; ^2^Department of Nursing, Seoul National University Hospital, Seoul, Republic of Korea; ^3^Department of Biomedical Science, Chosun University, Gwangju, Republic of Korea; ^4^BK FOUR Department of Integrative Biological Sciences, Graduate School of Chosun University, Gwangju, Republic of Korea; ^5^Institute of Well-aging Medicare & CSU G-LAMP Project Group, Chosun University, Gwangju, Republic of Korea; ^6^The Research Institute of Nursing Science, Seoul National University, Seoul, Republic of Korea

**Keywords:** cognitive function, generalized least square, interaction effects, longitudinal study, modifiability, older adults

## Abstract

**Background:**

Cognitive decline in older adults is influenced by diverse factors, and degrees of influence of these factors may vary depending on sex, age cohorts, and passage of time. Moreover, these factors differ in their responsiveness to general interventions. Thus, identifying these factors including their interactions with age, sex, and panel wave and conducting a systematic quantification of their influences on cognitive function are both necessary for developing efficient intervention strategies.

**Methods:**

To identify the influencing factors and their interactions, we applied a systematic stepwise variable selection using 2,535 community-dwelling older adults who participated in the Korean Longitudinal Study of Aging from Wave 5 (2014) to Wave 8 (2020). These factors were subsequently grouped based on their modifiability to investigate group-wise influences on cognitive function. For handling the longitudinal data, a generalized least squares method was used, and the degrees of influence of these factors were measured using the delta *R*^2^.

**Results:**

Twelve variables had significant main effects on cognitive function in older adults. Among these variables, age interacted with sex, regular exercise, and marital status. Sex interacted with regular exercise, education level, and depressive symptoms. Wave number interacted with depressive symptoms and social activity. In addition, the group-wise delta *R*^2^ values were found to be 10.9, 6.3, and 5.9% in the difficult-to-modify, modifiable, and non-modifiable factor groups, respectively. Afterwards, we provided the delta *R*^2^ for each sub-population divided by the levels of age, sex, and wave number to examine how these factors changed the influences.

**Conclusion:**

Based on the interaction and quantification results, we elucidated the characteristics of the influencing factors and their degrees of influence, and we suggest grouping factors based on their modifiability to systematically prevent cognitive decline in older adults.

## 1 Introduction

Cognitive decline is a natural process of human aging. As individuals age, their brains may undergo structural and functional changes, such as a decrease in volume and corresponding overactivity in the prefrontal cortex, as well as atrophy in the hippocampus (1). These changes are considered to be major causes of cognitive decline in older adults, and cognitive decline is a risk factor for mild cognitive impairment (MCI) or dementia, which subsequently reduces the quality of life (2).

From the perspective of public health, population aging is a global trend (3), and this is more pronounced in East Asian countries, especially South Korea. In 2022, 17.4% of the total population of South Korea was recorded as being aged 65 or older, and this percentage is expected to reach 47.7% by 2072 (4). Therefore, understanding the association between cognitive decline and influencing factors and developing appropriate strategies are critical concerns.

Although cognitive decline to a certain degree is inevitable in older adults, the progression rate of the symptoms can be controlled by various factors (5). Several studies have identified age, sex, education level, and marital status as socio-demographic factors (6, 7), smoking, drinking, exercise, and obesity as health behavioral factors (8, 9), and hypertension and diabetes mellitus as medical disease factors (8). Psychological factors including depression and anxiety (10, 11) and social factors such as social isolation (12) have also been shown to influence cognitive function.

The factors that influence cognitive function, as listed above, are both numerous and various in their characteristics. Therefore, when planning interventions to prevent cognitive decline in older adults, grouping factors with similar characteristics based on a criterion might be efficient. From this perspective, it is possible to consider the modifiability of these factors, because the criterion can be connected to susceptibility according to the types of intervention strategies. Factors with high modifiability generally include health behaviors or highly manageable symptoms. Therefore, these factors may be suitable for consideration as targets of regular interventions that can be applied to the whole population, regardless of individual characteristics. In contrast, factors with low modifiability include social statuses that are not easy to change, or relatively unmanageable symptoms. Therefore, they may be more appropriate for intervention targets for the corresponding specific sub-populations, and preemptive screening and managing may be more emphasized. To provide expected effects according to the differing intervention strategies, quantitative assessment of the degrees of influence in each factor group needs to be conducted in parallel with the grouping approach. Several studies have already emphasized the positive roles of modifiable factors on cognitive function (8, 9). However, while most of the previous studies have evaluated the influences of these factors through regression coefficients, to our knowledge, studies that have estimated their influences in terms of explained proportion of variation in cognitive function for classified factor groups have been relatively rare. Therefore, the present study may be the first to conduct a systematic and quantitative estimation of the influences of these factors or factor groups, categorized by their modifiability, on cognitive function in older adults.

For this purpose, we utilized data from the Korean Longitudinal Study of Aging (KLoSA), which contains longitudinal information on older adults in South Korea, to identify influencing factors and their interactions with the basic variables (i.e., age, sex, and KLoSA panel wave number) on cognitive function. Most previous studies focused on age (13) and sex differences (14, 15) in terms of interaction effects on cognitive function. However, in the present study, we additionally considered the panel “wave” variable to complement the age effect that may be confounded by the cohort effect. After identifying the influencing factors and their interactions, we estimated the influences of the individual factors and factor groups in both the whole population and sub-populations based on age, sex, and wave number. We expect that the results could serve as a basis for systematic and efficient intervention strategies for both the whole population and those at a higher risk for cognitive decline. For example, in South Korea, the Seoul Metropolitan Center for Dementia has implemented the Seoul Dementia Management Project (SDMP), a comprehensive community-based system for dementia care (16). The SDMP targets not only a high-risk group for dementia but also a normal cognition group, and it integrates early detection, case registration and management, and therapeutic interventions. This study may complement the SDMP by providing real data-based guidelines to the existing screening tools.

In summary, this study aimed to identify the factors influencing cognitive function in community-dwelling older adults aged 65 years or older in South Korea, using data from the KLoSA. More specifically, we utilized longitudinal data measured at four time points (from Wave 5 to Wave 8) to identify the factors influencing cognitive function in older adults and interactions of these factors with age, sex, and wave number. Subsequently, we categorized the factors based on their modifiability and quantifying the corresponding influences on cognitive function for comparison and interpretation.

## 2 Materials and methods

### 2.1 Study design

In this secondary data analysis study, we used data from the KLoSA to identify the factors that influence cognitive function in community-dwelling older adults aged ≥65 years and their interaction effects with age, sex, and wave number. Additionally, we calculated the degrees of influence of the individual factors or factor groups classified by their modifiability.

### 2.2 Study materials

This study used data from Waves 5 to 8 of the KLoSA (2014–2020), which was conducted by the Korea Employment Information Service (KEIS). The KLoSA provides nationally representative longitudinal data about Koreans aged ≥45 years, except for those living on Jeju Island, and uses a stratified multistage cluster sampling design based on region and types of housing. The panel was established with 10,254 individuals at Wave 1 (2006), is surveyed every 2 years, and is now open to Wave 8 (2020). Data were collected using computer-assisted personal interviewing (CAPI) method, in which the interviewer uses a laptop computer to read questions from the computer screen to the respondent and enter their responses (17).

**Figure 1:**
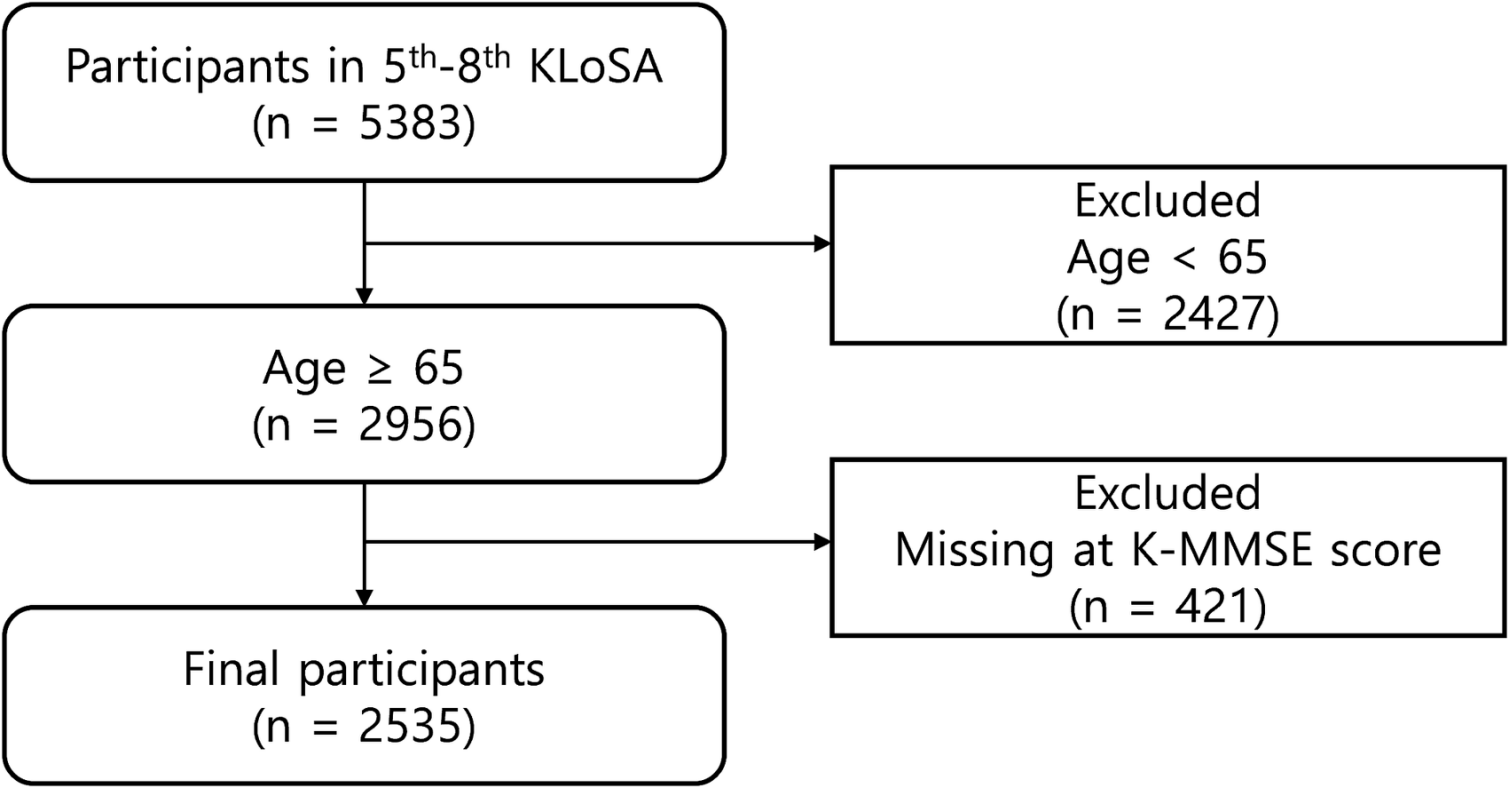
Flow chart of the filtering process of the study participants. KLoSA, Korean Longitudinal Study of Aging; K-MMSE, Korean Mini-Mental State Examination.

As shown in Figure 1, among the 5,383 participants in Wave 5 (2014), we selected those aged ≥65 years. Consequently, 2,427 participants were excluded. We additionally excluded 421 participants due to missing information regarding the cognitive function scores; thus, 2,535 participants were ultimately included in the analyses.

### 2.3 Study variables

#### 2.3.1 Socio-demographics

The socio-demographic variables used in the analysis were age (65–74 years as the young-old group or ≥ 75 years as the old-old group), sex (men or women), wave number (coded as 0,1,2 and 3 for 5^th^ to 8^th^ panel wave numbers, respectively), education level (elementary school or less, middle school, high school, and college or higher), and marital status (married or unmarried [single, separated, divorced, widowed, etc.]). Regarding the marital status variable, we combined single, separated, divorced, and widowed participants into the ‘unmarried’ category, as being single, separated, or divorced was rare in this study.

#### 2.3.2 Health behaviors

Regarding health behaviors, the following three variables were included: smoking, drinking, and regular exercise. The smoking variable was categorized as non-smoker, former smoker, or current smoker. A non-smoker is a person who has smoked fewer than 5 packs (100 cigarettes) in their lifetime and does not currently smoke. A person who does not currently smoke but has smoked at least 5 packs in their lifetime was classified as a former smoker. Lastly, a person who answered “Yes” to the question “Do you currently smoke?” was classified as a current smoker. Similarly, the drinking variable was categorized as non-drinker, former drinker, or current drinker. A non-drinker is a person who reported not drinking alcohol in both the previous and current surveys. A former drinker is a person who reported drinking alcohol at the time of the previous survey but no longer drinks alcohol at the time of the current survey. Lastly, a current drinker was defined as a person who answered “Yes” to the question “Do you occasionally or frequently drink alcohol?”. Regular exercise was dichotomized based on whether the participants exercised at least once a week.

#### 2.3.3 Health and disease status

In terms of health and disease status, we considered instrumental activities of daily living (IADL), body mass index (BMI), depressive symptoms, and several chronic disease statuses. IADL was measured according to 10 representative items by answering 0 for independence or 1 for dependence (18); thus, the total score may range from 0 to 10, with higher scores indicating lower levels in the function of daily living. BMI was calculated as weight [kg]÷height^2^ [m^2^] and categorized as underweight (<18.5), normal weight (≥18.5 and < 23), pre-obesity (≥23 and < 25), classI obesity (≥25 and < 30), and classII or severer obesity (≥30), referring the criteria of the 2022 BMI classification (19). Additionally, depressive symptoms were measured using the Center for Epidemiologic Studies Depression Scale (CES-D-10) Boston version (20), which is a shortened version of the CES-D with 20 items (21). The CES-D-10 has 10 items in total, and responses for the items are represented as integers from 0 (“I felt like that for a short time” or “I did not feel like that”) to 3 (“I felt like that all the time”) depending on how the participants had felt in the past week. Thus, the total score may range from 0 to 30, with higher scores indicating more severe depressive symptoms. Furthermore, regarding chronic disease statuses, hypertension, diabetes mellitus, coronary heart disease, and cerebrovascular disease were analyzed as binary variables according to the presence or absence of these diseases, based on self-reported information confirmed by a medical doctor’s diagnosis.

#### 2.3.4 Social activity

Social activity was operationally defined as the frequency of engagement in social groups. Accordingly, it was measured by the number of group activities in which the participants participated, including religious gatherings, social gatherings (e.g., senior centers), leisure/cultural/sports organizations (e.g., senior colleges), reunions/alumni, volunteer groups, and political parties/civic organizations. Since participation in more than two groups was rare in this study, we classified the numbers of participating groups into three categories: none, one group, and two or more groups.

#### 2.3.5 Cognitive function

Cognitive function was assessed using the Korean Mini-Mental State Examination (K-MMSE) (22), which was modified from the original MMSE (23) to account for cultural differences. In the K-MMSE, a total of six domains were tested: time orientation, place orientation, memory registration, memory recall, attention and calculation, and language and visual/spatial organization. The present study treated the total score as a continuous variable ranging from 0 to 30, with lower scores indicating a higher likelihood of cognitive impairment. When managing this as a categorical variable, three categories are generally used, with cutoff values of 18 and 24 (Table 1) (22, 24).

**Table 1:** Summary of the characteristics in the participants (*N* = 2,535).

Variables	Categories	*n* (%)^a^/M ± SD			
		5th	6th	7th	8th
Age, years	≥65 ~ ≤74	1,583 (62.4)	1,276 (50.3)	964 (38.0)	674 (26.6)
	≥75	952 (37.6)	1,259 (49.7)	1,571 (62.0)	1,861 (73.4)
Sex	Men	1,050 (41.4)			
	Women	1,485 (58.6)			
Education level^a^	Elementary school or less	1,492 (58.9)	1,490 (58.8)	1,489 (58.7)	1,490 (58.8)
	Middle school	394 (15.5)	393 (15.5)	394 (15.5)	394 (15.5)
	High school	471 (18.6)	473 (18.7)	473 (18.7)	472 (18.6)
	College or higher	178 (7.0)	179 (7.1)	179 (7.1)	179 (7.1)
Marital status	Unmarried	702 (27.7)	788 (31.1)	846 (33.4)	944 (37.2)
	Married	1,833 (72.3)	1,747 (68.9)	1,689 (66.6)	1,591 (62.8)
Smoking^a^	Non-smoker	1,808 (71.3)	1,795 (70.8)	1,783 (70.3)	1,775 (70.0)
	Former smoker	493 (19.4)	550 (21.7)	602 (23.7)	636 (25.1)
	Current smoker	234 (9.2)	190 (7.5)	150 (5.9)	124 (4.9)
Drinking^a^	Non-drinker	1,406 (55.5)	1,405 (55.4)	1,398 (55.1)	1,390 (54.8)
	Former drinker	382 (15.1)	439 (17.3)	535 (21.1)	634 (25.0)
	Current drinker	747 (29.5)	691 (27.3)	602 (23.7)	511 (20.2)
Regular exercise	Yes	859 (33.9)	887 (35.0)	838 (33.1)	991 (39.1)
	No	1,676 (66.1)	1,648 (65.0)	1,697 (66.9)	1,544 (60.9)
IADL (range 0–10)		0.38 ± 1.38	0.48 ± 1.62	0.59 ± 1.86	0.85 ± 2.30
BMI (kg/m^2^)^a^	<18.5	110 (4.3)	121 (4.8)	117 (4.6)	122 (4.8)
	≥18.5 ~ <23	1,069 (42.2)	1,079 (42.6)	1,083 (42.7)	1,083 (42.7)
	≥23 ~ <25	746 (29.4)	703 (27.7)	716 (28.2)	732 (28.9)
	≥25 ~ <30	576 (22.7)	603 (23.8)	585 (23.1)	560 (22.1)
	≥30	34 (1.3)	29 (1.1)	34 (1.3)	38 (1.5)
Hypertension		1,326 (52.3)	1,400 (55.2)	1,477 (58.3)	1,538 (60.7)
Diabetes mellitus		546 (21.5)	597 (23.6)	652 (25.7)	703 (27.7)
Coronary heart disease		295 (11.6)	324 (12.8)	344 (13.6)	381 (15.0)
Cerebrovascular disease		162 (6.4)	196 (7.7)	221 (8.7)	246 (9.7)
CES-D-10 (range 0–30)		4.96 ± 5.05	4.67 ± 4.89	5.20 ± 5.10	4.81 ± 4.67
Social activity	0	660 (26.0)	764 (30.1)	754 (29.7)	1,102 (43.5)
	1	1,445 (57.0)	1,341 (52.9)	1,439 (56.8)	1,157 (45.6)
	≥2	430 (17.0)	430 (17.0)	342 (13.5)	276 (10.9)
K-MMSE (range 0–30)^a^		24.16 ± 5.39	23.99 ± 5.39	23.29 ± 6.05	22.48 ± 6.21
	≤17	311 (12.3)	328 (12.9)	418 (16.5)	502 (19.8)
	≥18 ~ ≤23	638 (25.2)	666 (26.3)	694 (27.4)	757 (29.9)
	≥24	1,586 (62.6)	1,541 (60.8)	1,423 (56.1)	1,276 (50.3)

a Totals may not equal 100% because of rounding.

BMI, Body mass index; CES-D-10, The 10-item Center for Epidemiologic Studies of Depression Scale; IADL, Instrumental activities of daily living; K-MMSE, Korean Mini-Mental State Examination; M, Mean; SD, Standard deviation.

### 2.4 Data collection procedures

The KLoSA provides de-identified data that can be downloaded from the KEIS website.^1^ There was no approval process other than registration, and we freely downloaded and utilized the raw data, questionnaires, and codebooks.

### 2.5 Data analysis

All statistical analyses were conducted using R version 4.3.2. We used descriptive statistics such as frequencies, percentages, means, and standard deviations to explore the general characteristics of the study participants.

We then constructed a model of the factors influencing cognitive function using the generalized least squares (GLS) method. The GLS model is an extension of linear regression (22), which is suitable for analyzing repeatedly measured data by allowing correlations between errors. Since the KLoSA conducts a panel survey every 2 years, it was assumed that the covariance matrix of the errors had an autocorrelation structure. In our analysis, we used a stepwise selection process to systematically select the significant variables and their interactions in the GLS model. Before stepwise selection, age, sex, and wave number were fixed as the basic variables. Subsequently, we selected other significant variables as the main effects and included significant interaction effects between the selected and basic variables. In this step, we first tested the second-order interaction and then examined the third-order interaction using the previously selected second-order interaction and remaining basic variables. During the stepwise process, when an interaction effect was significant, the main or lower-order interaction terms contained in the interaction term were not excluded from the model, irrespective of their significance.

Finally, to systematically estimate and compare the degrees of influence of the factors on cognitive function, we used delta *R*^2^, which indicates the difference in the *R*^2^ values between the models with and without a variable of interest. For variables that had interaction effects, the delta *R*^2^ was calculated as the difference in the *R*^2^ values between the models with and without all terms containing the variable of interest. The delta *R*^2^ was calculated for the following three targets: (1) individual factors in the model with the total population, (2) factor groups in the model with the total population, and (3) factor groups in the model with sub-populations divided by age, sex, and wave number. The categories of the factor groups were as follows: (1) non-modifiable factor group (i.e., basic variables), (2) difficult-to-modify factor group, and (3) modifiable factor group.

Regarding imputation, there were missing data in height, weight, and depressive symptom variables. For the height and weight variables, missing data were present in <3% in each wave, and these were imputed using the mean of the observed information of the same participants in the other waves, considering that these variables are generally stable over time. When a participant had missing data in all waves, we imputed the data using the mean of the observed information of other participants with identical age and sex. Regarding the depressive symptom variable, only two participants had missing data at one wave each, and we imputed these using the mean of the scores in the other waves. *p*-values under 0.05 were considered statistically significant for all analyses.

### 2.6 Ethical considerations

This study received an exemption from the Institutional Review Board of Seoul National University (No. E2405/004-003).

## 3 Results

### 3.1 General characteristics of the participants

The general characteristics of the selected participants are shown in Table 1. In the socio-demographic variables at Wave 5, the majority were the young-old (62.4%), women (58.6%), educated to elementary school or less (58.9%), and married (72.3%).

In terms of health behaviors, 71.3% were non-smokers and 55.5% were non-drinkers in Wave 5. Notably, the proportion of current smokers decreased from 9.2% at Wave 5 to 4.9% at Wave 8, whereas the proportion of current drinkers decreased from 29.5 to 20.2% over the same period. In addition, nearly two out of three participants (66.1%) did not exercise regularly (at least once a week) at Wave 5.

Regarding health and disease status, the mean IADL scores gradually increased from 0.38 ± 1.38 at Wave 5 to 0.85 ± 2.30 at Wave 8, indicating that the overall ability to perform daily living activities tended to decrease over time. Concerning the BMI variable, the normal weight range was the most prevalent (42.2%) of the five BMI classifications among the participants at Wave 5. Furthermore, regarding chronic disease statuses, 52.3% of the participants had hypertension, 21.5% had diabetes mellitus, 11.6% had coronary heart disease, and 6.4% had cerebrovascular disease. Notably, all the aforementioned chronic diseases tended to steadily increase in prevalence over time. In contrast, the mean depressive symptom score was 4.96 ± 5.05 at Wave 5 and had no clear trend in the wave variable. In terms of social activity, more than half (57.0%) participated in one social activity. Finally, regarding cognitive function, the mean K-MMSE score gradually decreased from 24.16 ± 5.39 at Wave 5 to 22.48 ± 6.21 at Wave 8.

### 3.2 Identification and statistical interpretation of the influencing factors on cognitive function and their interactions in older adults

Upon stepwise selection of significant variables in the GLS model (Table 2) in addition to the three basic variables (age, sex, and wave number), we identified the following variables as main effects: education level, marital status, drinking, regular exercise, IADL, BMI, cerebrovascular disease, depressive symptoms, and social activity. The following second-order interaction terms were significant: age × sex, age × marital status, sex × education level, sex × depressive symptoms, wave × depressive symptoms, and wave × social activity. As the third-order interaction term, age × sex × regular exercise had significant effects.

**Table 2:** The generalized least squares regression results on cognitive function, including the main and interaction effects.

Variables	Categories	*B*	SE	*t*	*p*–value	Anova *p*	FDR *q*
Main effects							
Intercept		24.67	0.41	60.45	<0.001	–	–
Age, years (ref. ≥65 ~ ≤74)	≥75	−1.96	0.35	−5.63	<0.001	–	–
Sex (ref. Men)	Women	−0.86	0.28	−3.08	0.002	–	–
Wave		−0.46	0.08	−5.94	<0.001	–	–
Education level (ref. Elementary school or less)	Middle school	1.12	0.28	3.96	<0.001	<0.001	<0.001 (<0.001)
	High school	1.69	0.25	6.79	<0.001		
	College or higher	2.47	0.32	7.84	<0.001		
Marital status (ref. Unmarried)	Married	0.12	0.19	0.65	0.519	–	0.751 (0.197)
Drinking (ref. Non–drinker)	Former drinker	−0.51	0.18	−2.85	0.004	<0.001	<0.001 (<0.001)
	Current drinker	0.14	0.18	0.77	0.442		
Regular exercise (ref. No)	Yes	0.61	0.16	3.78	<0.001	–	<0.001 (<0.001)
IADL (range 0–10)		−0.72	0.03	−28.36	<0.001	–	<0.001 (<0.001)
BMI (kg/m^2^) (ref. <18.5)	≥18.5 ~ <23	0.10	0.25	0.38	0.703	<0.001	<0.001 (<0.001)
	≥23 ~ <25	0.57	0.26	2.18	0.030		
	≥25 ~ <30	0.62	0.27	2.28	0.023		
	≥30	0.29	0.50	0.59	0.556		
Cerebrovascular disease (ref. No)	Yes	−1.24	0.22	−5.64	<0.001	–	<0.001 (<0.001)
CES–D–10 (range 0–30)		−0.09	0.02	−5.16	<0.001	–	<0.001 (<0.001)
Social activity (ref. 0)	1	0.71	0.16	4.53	<0.001	<0.001	<0.001 (<0.001)
	≥2	0.89	0.22	4.10	<0.001		
Second order interaction effects							
Age, years (≥75) × Sex (Women)		−1.44	0.33	−4.33	<0.001	–	<0.001 (<0.001)
Age, years (≥75) × Regular exercise (Yes)		0.02	0.27	0.07	0.948	–	1.000 (0.476)
Age, years (≥75) × Marital status (Married)		0.66	0.28	2.34	0.019	–	0.125 (0.011)
Sex (Women) × Education level (Middle school)		0.50	0.38	1.30	0.193	0.017	0.125 (0.009)
Sex (Women) × Education level (High school)		0.83	0.38	2.23	0.026		
Sex (Women) × Education level (College or higher)		1.72	0.66	2.60	0.009		
Sex (Women) × CES–D–10		−0.05	0.02	−2.43	0.015	–	0.125 (0.006)
Sex (Women) × Regular exercise (Yes)		−0.34	0.22	−1.56	0.120	–	0.566 (0.034)
Wave × CES–D–10		−0.02	0.01	−2.26	0.024	–	0.132 (0.014)
Wave × Social activity (1)		0.27	0.08	3.49	0.001	<0.001	0.008 (<0.001)
Wave × Social activity (≥2)		0.36	0.11	3.18	0.002		
Third order interaction effects							
Age, years (≥75) × Sex (Women) × Regular exercise (Yes)		1.22	0.37	3.29	0.001	–	0.012 (0.001)

False discovery rates (FDR) are additionally provided based on the original (outside the parentheses) (60) and sequential FDR (inside the parentheses) method (61) in the last column.

B, Unstandardized coefficients; BMI, Body mass index; CES-D-10, The 10-item Center for Epidemiologic Studies of Depression Scale; FDR, False discovery rate; IADL, Instrumental activities of daily living; SE, Standard error.

**Figure 2:**
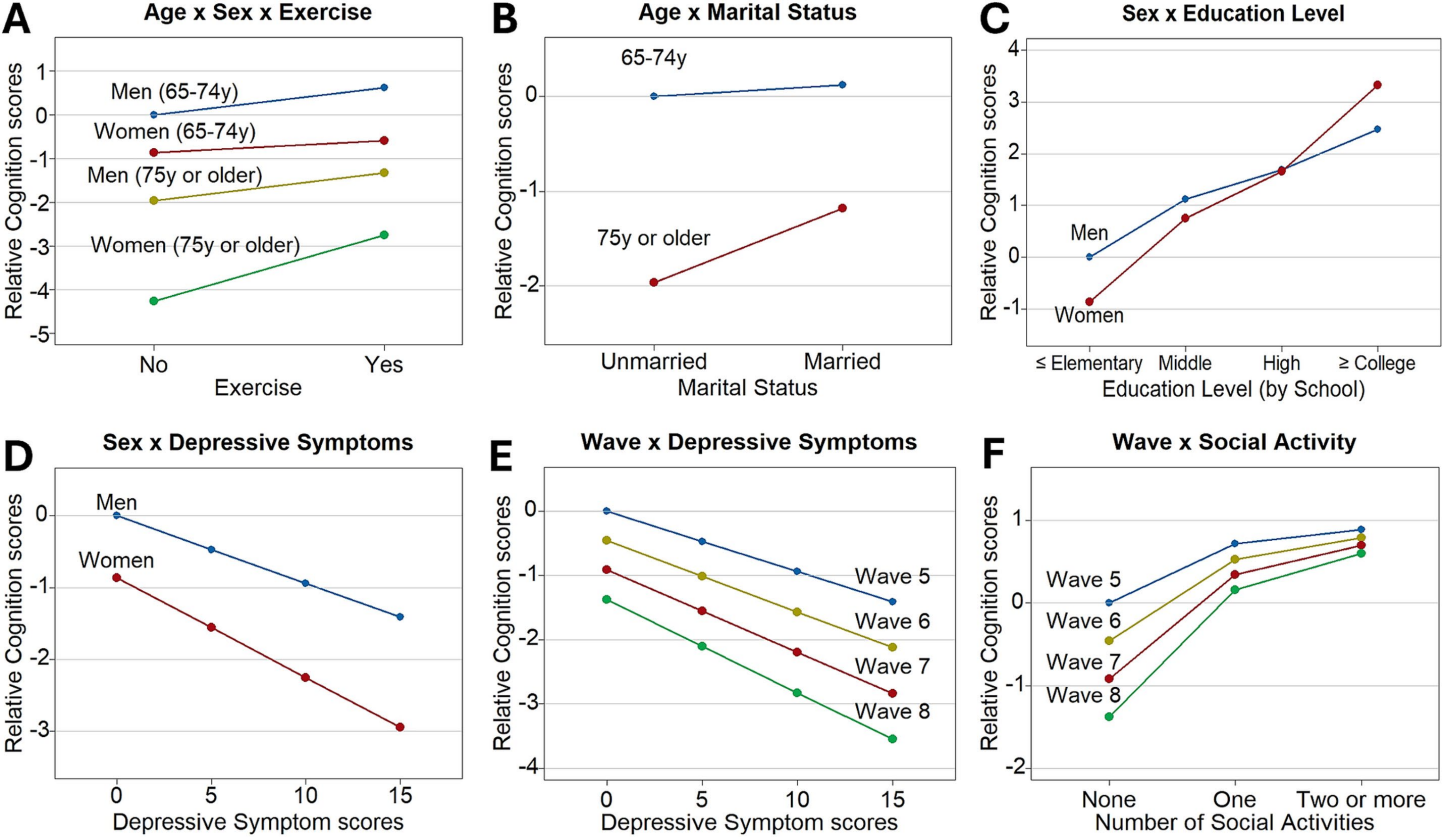
The significant interactions of the influencing factors with age, sex, and wave number on cognitive function. **(A)** The effect of regular exercise interacted with age and sex. **(B)** The effect of marital status interacted with age. **(C)** The effect of education level interacted with sex. **(D)** The effect of depressive symptoms interacted with sex. **(E)** The effect of depressive symptoms interacted with wave number. **(F)** The effect of social activity interacted with wave number. In the *y*-axis, the relative cognition scores mean the distances in the cognitive function scores from those at the baseline levels.

Using the results of this association analysis, plots were drawn to show the specific interactions (Figure 2). First, we focused on age-related interactions. Regarding the third-order interaction (Figure 2A), we interpreted the main effects of these three factors in advance. As shown in Table 2, the old-old group (*B* = −1.96, *p* < 0.001) and women (*B* = −0.86, *p* = 0.002) had negative effects on cognitive function, whereas regular exercise (*B* = 0.61, *p* < 0.001) had a positive effect compared to their reference groups. In terms of the second-order interactions, the significant negative age × sex effect (*B* = −1.44, *p* < 0.001) indicated that the old-old women in the non-exercising group showed a greater decline in cognitive function. However, the significant age × sex × regular exercise effect (*B* = 1.22, *p* = 0.001) showed that regular exercise was associated with cognitive decline in old-old women. Figure 2A shows that regular exercise was associated with a slight increase (0.28 points) in cognitive function among the young-old women, whereas a substantial increase (1.51 points) was observed in the old-old women. Age also interacted with marital status (*B* = 0.66, *p* = 0.019), with the “married” status having a more positive effect on cognitive function in the old-old group than in the young-old group. Figure 2B visualizes this interaction effect, showing that the effect of the marital status was relatively small for the young-old group (0.12) but high for the old-old group (0.79).

Second, we focused on the sex-related interactions. We found that sex interacted with both the education level and depressive symptom score. In the case of education level, we found a positive association with cognitive function. Specifically, the middle school (*B* = 1.12, *p* < 0.001), high school (*B* = 1.69, *p* < 0.001), and college or higher (*B* = 2.47, *p* < 0.001) education levels were positively associated with cognitive function in men when compared with the group educated to the elementary school or less. In contrast, the depressive symptom score was negatively associated with cognitive function in men (*B* = −0.09, *p* < 0.001). However, both the education level and depressive symptom scores had enhanced effects in women, regardless of their directions. In terms of the education level, the additional interaction effects in women were positive for all levels of education, with especially significant effects in the high school (*B* = 0.83, *p* = 0.026) and college or higher (*B* = 1.72, *p* = 0.009) education levels. Consequently, the positive interaction effects of the education level were high enough to compensate for sex differences at the college or higher education level (Figure 2C). Regarding the sex × depressive symptoms effect, each 1-point increase in the depressive symptom score in women corresponded to an additional 0.05-point decrease in the cognitive function score (*B* = −0.05, *p* = 0.015). Therefore, decreases in cognitive function were steeper in women (−2.09) than in men (−1.41) as the depressive symptom scores increased from 0 to 15 (Figure 2D).

Third, we focused on the wave-related interactions, and found that the wave variable interacted with both the depressive symptom score and social activity. The wave number was negatively associated with cognitive function (*B* = −0.46, *p* < 0.001) when the two interacting variables were at the reference levels. In the case of the depressive symptom score, we already confirmed the negative effect in the above (*B* = −0.09, *p* < 0.001). However, participation in social activities was positively associated with cognitive function (one social activity: *B* = 0.71, *p* < 0.001; two or more social activities: *B* = 0.89, *p* < 0.001) compared with the group with no social activity. These two effects, estimated at Wave 5, were significantly enhanced with the increasing waves. Specifically, the depressive symptom score had an additional negative effect for each increase in the wave number (*B* = −0.02, *p* = 0.024). Figure 2E shows the increasing negative effects of the depressive symptom score on cognitive decline as the wave increases. Similarly, an increased number of social activities had additional positive effects per increase in the wave number (one social activity: *B* = 0.27, *p* = 0.001; two or more social activities: *B* = 0.36, *p* = 0.002). As a result, cognitive function scores slightly increased with more social activities compared to no social activity in Wave 5 (one social activity: 0.71; two or more social activities: 0.89), but the increase became more substantial as wave increased (at Wave 8, one social activity: 1.53; two or more social activities: 1.97) (Figure 2F).

In this analysis, the variables that only had main effects were drinking, IADL, BMI, and cerebrovascular disease. Notably, the pre-obesity (*B* = 0.57, *p* = 0.030) and classI obesity (*B* = 0.62, *p* = 0.023) groups maintained a significantly higher cognitive function than the underweight group (Table 2). Additionally, the participants with a former drinking status (*B* = −0.51, *p* = 0.004), higher IADL score (*B* = −0.72, *p* < 0.001), or cerebrovascular disease (*B* = −1.24, *p* < 0.001) had significantly lower cognitive function than the participants at the reference levels of the variables.

### 3.3 Systematic comparing the degrees of influence of the factors on cognitive function in older adults

Table 3 presents the degrees of influence of the factors based on the results of the GLS model. First, when we sorted the individual factors by descending order of the delta *R*^2^, IADL (5.8%), age (5.0%), education level (3.5%), and depressive symptom score (2.9%) had values >2%. The total *R*^2^ of the model with the total population was 37.7%.

**Table 3:** The delta *R*^2^ of the individual factors and the factor groups.

Factors	Delta *R*^2^ (%) in the total population^a^				Delta *R*^2^ (%) in each sub-population^a^							
	Individual factors	Factor groups			Age (65 ~ 74y)				Age (75y~)			
					Men		Women		Men		Women	
					5th	8th	5th	8th	5th	8th	5th	8th
Age	5.0	5.9	25.4	5.9	NA							
Sex	1.3											
Wave	0.3											
Education level	3.5	10.9		20.2	8.4	11.3	4.4	9.1	13.5	17.0	9.9	15.7
Marital status	0.2											
Cerebrovascular disease	0.5											
IADL	5.8											
BMI	0.3	6.3	6.3		10.3	10.7	9.4	12.7	8.7	8.2	9.7	10.9
CES-D-10	2.9											
Drinking	0.2											
Regular exercise	0.7											
Social activity	1.7											
Total	37.7				27.7	37.0	20.3	33.6	31.8	45.3	29.6	42.0

The factors were grouped according to the degrees of modifiability; The delta *R*^2^ for the factor groups were calculated from the results with the total population and sub-populations divided by the basic variables, respectively.

a The delta *R*^2^ was calculated with the difference between the model’s *R*^2^ with and without the specific factors or factor groups.

BMI, Body mass index; CES-D-10, The 10-item Center for Epidemiologic Studies of Depression Scale; IADL, Instrumental activities of daily living; NA, Not available.

Second, we classified the influencing factors based on their modifiability, and evaluated the delta *R*^2^ in terms of the factor groups. As a result of the classification, age, sex, and wave number were grouped as non-modifiable factors. In the difficult-to-modify factor group, education level, marital status, cerebrovascular disease status, and IADL were contained. For the modifiable factor group, BMI, depressive symptoms, drinking, regular exercise, and social activity were included. When we calculated and sorted the delta *R*^2^ values by descending order, the values were as follows: 10.9% in the difficult-to-modify factor group, 6.3% in the modifiable factor group, and 5.9% in the non-modifiable factor group. When the above three groups were combined and reclassified into two groups, the delta *R*^2^ of the group including non-modifiable and difficult-to-modify factors was 25.4%, and that of the group including difficult-to-modify and modifiable factors was 20.2%.

Finally, Table 3 shows how the delta *R*^2^ values in the modifiable and the difficult-to-modify factor groups changed according to the levels of the basic variables. For this purpose, we divided the total population into eight sub-populations based on a combination of the levels of the three basic variables (two levels in each group of age and sex, and Wave 5 and Wave 8). We observed the following patterns from the factor group-wise delta *R*^2^ for each sub-population. First, regarding age, the delta *R*^2^ for the difficult-to-modify factor group was consistently higher in the old-old group than in the young-old group when the levels of the other basic variables were fixed. In contrast, the delta *R*^2^ for the modifiable factor group was generally lower in the old-old group than in the young-old group. Thus, the young-old group showed a relatively higher delta *R*^2^ for the modifiable factor group, whereas the old-old group showed a higher delta *R*^2^ for the difficult-to-modify factor group. Second, in terms of sex, the delta *R*^2^ of the difficult-to-modify factor group was generally higher in men than in women, whereas the delta *R*^2^ of the modifiable factor group was similar, or higher in women than in men. Third, concerning the wave number, the increases of the delta *R*^2^ from Wave 5 to Wave 8 in the difficult-to-modify factor group were higher than those in the modifiable factor group. Finally, the overall *R*^2^ in the sub-populations tended to be higher in the old-old group than in the young-old group, higher in men than in women, and higher in Wave 8 than in Wave 5.

As an additional analysis, to investigate the subgroup-specific influences without the IADL variable which can sometimes be regarded as a result of cognitive decline, GLS regression (Supplementary Table 1) and the corresponding delta *R*^2^ calculation (Supplementary Table 2) were conducted again without the variable. In Supplementary Table 1, the association results of the other influencing factors generally remained consistent. However, as shown in Supplementary Table 2, the exclusion of IADL resulted in generally sharp decreases of delta *R*^2^ of the difficult-to-modify factor group and notable increases in delta *R*^2^ of the modifiable factor group.

## 4 Discussion

This study was conducted to systematically identify the factors that influence cognitive function in community-dwelling older adults in South Korea and their interactions with age, sex, and panel wave using longitudinal data measured at four time points. One of the notable strengths of our study is that we additionally introduced delta *R*^2^ to measure explained proportion of variation of cognitive function for both individual factors and factor groups. The factor groups were constructed based on the modifiability of the individual factors. Based on these results, we could provide a framework for efficient intervention strategies by prioritizing the influencing factors or factor groups according to subgroups.

Interestingly, smoking was not selected as a significant main effect variable. There were some controversies about the effects of smoking on cognitive function (25–27). The controversies might come from not only heterogeneity of study populations but also from how the variable was categorized. Therefore, further studies focusing on the smoking variable are needed.

We selected age, sex, and wave number as targets for the interactions because they are not only representatively basic for distinguishing a population, but also purely exogenous. Of these variables, both age and wave number are associated with time. However, they differ in their conceptual roles. The age variable might be confounded by cohort effects between the groups, as it also captures inter-individual (between-person) variations arising from generational differences. In contrast, the wave variable primarily reflects intra-individual (within-person) differences in the aging process that was equally applied to all participants over the same period. The findings in this study also support the need for distinction, since age interacted with sex, regular exercise, and marital status, whereas wave number interacted with depressive symptoms and social activity.

Among the interactions with the three basic variables, we first focused on interactions with age. We found that the old-old group had a lower cognitive function score than the young-old group when the other interacting variables were at the baseline levels. These results could be interpreted as the natural causal effect of aging (1); however, we found that the effects of age were moderated by sex and regular exercise. The negative interaction between age and sex implies that the difference of cognitive function between men and women became larger in the old-old group than in the young-old group. In terms of biology, this may be because women typically have smaller gray matter than men (28); therefore, women are more susceptible to aging and neurodegenerative diseases (29).

In the case of regular exercise, the positive effect on cognitive function in older adults has already been supported by previous studies (30, 31). Although aging is a common cause of declining exercise capacity, exercise efficiency could be higher in older adults than in young adults from exercise training program interventions (32). Regarding exercise efficiency, a previous meta-analysis (33) found that older women had a higher exercise efficiency than older men, and these results supported our findings of third-order interaction. In addition, a previous study (34) emphasized the importance of exercise interventions for old-old women by showing that a twice-weekly exercise program intervention significantly improved memory, attention, calculation, and language functions in the group. When providing exercise interventions for the old-old women, it is essential to consider their age- and gender-specific characteristics. As individuals age, they become more prone to musculoskeletal strain, and women are generally more susceptible to psychological conditions such as depression (35). Therefore, to help the old-old women maintain regular exercise, low-intensity exercises that reduce musculoskeletal strain and incorporate social interactions (e.g., group fitness classes) can be highly effective interventions.

In the present study, age also interacted with marital status. We found that the married group generally had a higher cognitive function than the group without a spouse, which is consistent with the findings of a previous study (36). Considering that married people have many advantages in terms of receiving additional emotional support (37) and a positive influence on health and lifestyle from their spouses (38), being married may contribute to protecting against cognitive decline. In addition, the advantage of being married was significantly enhanced in the old-old group. A previous study (39) found that although the changing trend of cognitive function with age had a steeply declining curve, the slope in the married group was more gradual than that in the widowed group. Briefly, the difference in cognitive function between the two groups is more pronounced in the old-old group compared to the young-old group. Therefore, we suggest the proactive screening and management of older adults without a spouse.

Regarding the sex variable, the mean score of cognitive function in women was lower than that in men when the other interacting variables were at the reference levels. However, the positive effects of education in women were larger than those in men. These results were consistent with those reported in a previous study (40) analyzing the effects of education levels on cognitive function in older Korean Americans, which also found that higher educational levels resulted in greater positive effects on cognitive function in women than in men (40). This might indicate that the sex differences in cognitive function can be compensated by interventions concerning sociological resources.

Such sex-specific education effects can be interpreted in terms of the Resource Substitution theory (41), which implies that when women have low socioeconomic resources compared to men, the supply of education acts as a greater catalyst for improved health in women than in men (41). In other words, this theory emphasizes that the positive effect of education on health is greater for women than for men. In fact, in South Korea, women in the older generation have experienced social inequalities due to less access to socioeconomic resources such as authority, power, and capital when compared to men (42). According to a Korean national report (43), the proportion of older men with high school or higher education is 47.8%, which is nearly twice that of older women (=24.3%), implying that access to higher education for women was also limited in the past generations. However, based on the Resource Substitution theory, education for women with these inequalities may result in greater benefits regarding cognitive function when compared to men. In addition, since we used more detailed levels of education than the previous study (40), which used a binary variable, we were able to observe that the mean cognitive function was higher in women than in men at the college or higher education level.

In the case of depressive symptoms, the association and the corresponding mechanisms between depression and cognitive decline has already been established through numerous studies (44, 45). In addition, the interaction between sex and depressive symptoms can be explained by the sex-specific hormonal change in postmenopausal women (46). In detail, the previous study reported that the depressive disorder at the baseline was associated with a higher risk of developing MCI or dementia in postmenopausal women aged 65–79 years (46). This might be due to decreased estrogen levels, which induce deficits in memory functions (47). However, another study (48) reported conflicting results of a stronger relationship in older men. These inconsistencies might have been caused by the different methodologies of these studies. As in the aforementioned studies (46, 48), numerous studies have treated depressive symptoms as a binary variable, with inconsistent cutoffs in the variables; therefore, analysis using the binary variable may induce both a loss of information and conflicting results. In contrast, a previous study (49) analyzed this interaction effect with more detailed categories, and found a stronger effect in women experiencing moderate-to-severe symptoms. In the present study, we used the depressive symptom scores as a continuous variable, as it provides more precise results for the interaction effect; however, further studies are required to draw clearer conclusions regarding this interaction effect.

Finally, the third basic variable, the panel wave number, negatively affected cognitive function when the levels of the other interacting variables were at baseline. In terms of interactions, the wave number and depressive symptoms mutually enhanced their negative effects. To interpret this interaction effect, we suggest two possible explanations from the existing research. First, both human aging (1) and depressive symptoms (45) can be explained by similar biological mechanisms such as a reduction in the hippocampal volume. Second, considering that the slope of cognitive decline becomes steeper as age increases (39), it is reasonable to speculate that there may be an additional effect on the sum of the linear main effects in the wave number and depressive symptoms.

However, the interaction effect between the wave number and social activity implies that cognitive decline induced by aging can be related to participation in social activities. The positive effect of social activity has already been confirmed in a previous study (50), which showed that higher social isolation was associated with lower test scores in composite cognitive function, verbal fluency, and forward digit span. This interaction effect was also similar to that shown in a previous 12-year follow-up study (51), wherein the decline of global cognitive function in participants with frequent social activities (top 10%) was only 30% of that in those with infrequent social activities (bottom 10%) (51). According to a survey (52) on whether people have someone to rely on in difficult times, the response rates for ‘No’ in Germany, the United States, and Japan were within 5–12%, whereas in South Korea, the rates often exceeded 20%. These results imply that demand for interventions targeting social isolation may be higher in South Korea than in the aforementioned countries, emphasizing the critical need for programs that promote social participation. Moreover, since the effect was much larger between the zero and one social activity groups than between the one and two or more social activity groups, it is crucial to encourage at least one social activity where interventions are planned against cognitive decline in older adults.

Thus far, we have focused on the interpretations of the individual influencing factors and their interactions with age, sex, and wave number. We also interpreted the results in terms of the factor groups for planning specific intervention strategies. Among the groups, we first focused on the modifiable factors because interventions targeting these factors are easier to conduct and expected to be more effective than those targeting the difficult-to-modify factors. Consequently, the modifiable factors should be prioritized among the targets for intervention strategies. Biologically, such interventions have been found to alleviate cognitive impairment by regulating cognitive reserve, which is defined as an individual’s ability to cope with a decline in brain function (53). In other words, interventions for modifiable factors may reduce cognitive impairment by increasing brain resilience (54); this can be regarded as a multidomain intervention (55), which has greater effects than a single-domain intervention.

Second, the difficult-to-modify factor group had a higher delta *R*^2^ than the modifiable factor group; however, interventions targeting these factors are difficult to conduct and restricted to specific sub-populations. This implies that they require more precise targeting for the corresponding participants. Moreover, considering the low modifiability in the group, preemptive identification and management of these individuals is more critical than post-management. However, although education level, IADL, marital status, and cerebrovascular disease were included in the difficult-to-modify factor group, this did not mean that they could not be improved or changed. For example, exposure to intellectual activity is associated with the improvement and maintenance of IADL (56). Hence, the negative effects of a lower education level and higher IADL score on cognitive function could be alleviated by providing interventions in the form of intellectual activities, such as the regular reading of newspapers or books. Similarly, social isolation related to marital status may be relieved by providing interventions in the form of home-visiting (57).

Based on this, the modifiable and difficult-to-modify factors may have a mutually influential relationship. The increased influences of the modifiable factor group observed in the analysis excluding IADL suggests that the influence of the IADL variable may have been absorbed into the modifiable factor group. This may be because IADL intrinsically reflects an individual’s capacity to perform daily activities through its associations with modifiable factors such as social activity and regular exercise. Furthermore, if modifiable factors concerning lifestyle are not properly managed, they may lead to difficult-to-modify factors, such as cerebrovascular diseases (58). Therefore, interventions targeting modifiable factors not only provide direct benefits to cognitive function but also indirectly contribute to the improvement or maintenance of difficult-to-modify factors. In the case of education level, which demonstrated the third highest delta *R*^2^ value, this factor may have a higher modifiability for future generations. Therefore, more active policy-level approaches such as providing continuous learning opportunities and social and economic support for future generations should be considered to efficiently prevent cognitive decline.

Third, for the non-modifiable factor group, the delta *R*^2^ was the smallest (5.9%) among those in the three groups, and much smaller than that of the total model (37.7%). This implies that the degree of cognitive decline would mainly depend on efforts to conduct appropriate interventions based on the other two factor groups.

Finally, to complement the group-wise interpretations of the association results, we also provided the delta *R*^2^ for sub-populations divided by age, sex, and wave number. Notably, we found that the influences on cognitive function in the difficult-to-modify factor group increased with age or wave number. In contrast, the influences in the modifiable factor group increased relatively less or even decreased in general with age or wave number. Therefore, the modifiable factor group had a higher influence on cognitive function in the young-old group, whereas the difficult-to-modify factor group had a higher influence in the old-old group. Consequently, for modifiable factors, early interventions targeting the young-old group are prioritized to maximize long-term effects, such as encouraging regular exercise and social interactions. On the other hand, for difficult-to-modify factors, proactive strategies are essential for the old-old group, where the impact of these factors on cognitive function becomes more pronounced with age. Such strategies also include screening older adults at high risk for these factors, and providing appropriate personalized support.

In terms of sex, the influences in the difficult-to-modify factor group were relatively higher in men than in women, whereas the modifiable factor group showed the opposite trend. It can be interpreted that difficult-to-modify factors tend to have stronger and earlier influences on men than women. Furthermore, susceptibility to the intervention and the corresponding effects might differ according to sex. Consequently, sex-specific approaches may need to be considered. Specifically, early and regular diagnosis and intervention programs, such as cognition tests and chronic disease prevention and management programs, may be more effective for older men than for older women. In the case of older women, they are relatively more affected by the modifiable factors than men. Considering that the negative effects of the modifiable factors may be accumulated with the passage of time (59), medium- to long-term management and monitoring strategies are required.

In summary, this study is meaningful in that it systematically explored the influencing factors and their interactions to the third-order through a stepwise selection approach. In addition, we classified these factors according to their potential for modifiability, and quantitatively compared their influences on cognitive function to provide a basis for planning intervention strategies. Despite the strengths of this study, it has the following limitations. First, although the survey was conducted to minimize bias by training interviewers with CAPI, the KLoSA data is basically a self-report survey, which may be subject to response bias. Future studies may improve accuracy by involving family members or caregivers in the interview. Second, while the K-MMSE tool has been validated and popular, it is also a screening tool. Hence, we suggest that future studies may need to include a clinical diagnosis of cognitive impairment through imaging tools such as magnetic resonance imaging. These additional approaches will provide a more comprehensive evaluation by integration with objective data on structural and pathological changes in the brain (e.g., brain atrophy, gray matter volume reduction). Third, although the age and panel wave variables were clearly distinguished in terms of statistical modeling and the association results were different between them, these variables overlap to a certain degree in terms of clinical interpretation, because they share the concept of aging. Therefore, interpretation and application may need to be confirmed with additional investigations. Fourth, the findings of this study, which are based on a Korean population, may have limited generalizability to other populations due to cultural, social, and demographic differences. Fifth, although we considered several aging-related disease statuses as shown in Table 1, it may not be sufficient, and the KLoSA does not include other aging-related factors, such as metabolic factors and inflammatory biomarkers. Consequently, the effect of the age variable can be overestimated by absorbing these unobserved effects in terms of regression coefficient and delta *R*^2^.

## 5 Conclusion

This study first focused on the exploration of factors that influence cognitive function in community-dwelling older adults in South Korea and their interactions with age, sex, and the KLoSA panel wave number. We constructed a model for cognitive function in this population, including various factors such as socio-demographics, health behavior, health and disease status, and social factors. From the analysis of the longitudinal data and the identified interactions, we could understand the characteristics of the subgroups in more detail according to the passage of time. Moreover, through classifying these factors based on their modifiability and quantifying them with delta *R*^2^, we could estimate the effects of these factor groups. We expect that these tailored results can be used for planning targeted and efficient intervention strategies by prioritizing the influencing factors or factor groups according to subgroups. Based on the findings of this study, we propose that future intervention policies should more actively adopt quantitative analysis results for more systematic and tailored applications. Specifically, the insights from this study could provide real data-based guidelines for prioritizing influencing factors and the corresponding interventions according to subgroups in the design and implementation of the social welfare system (e.g., the SDMP) for older adults.

## Data Availability

Publicly available datasets were analyzed in this study. This data can be found here: https://survey.keis.or.kr/eng/klosa/klosa01.jsp.

## Footnotes

^1^ https://survey.keis.or.kr/eng/klosa/klosa01.jsp
