# Supplementary material for: Systematic identification and quantification of factors and their interactions with age, sex, and panel wave influencing cognitive function in Korean older adults
Source: Front Public Health. 2025 Feb 3;13:1547575. doi: 10.3389/fpubh.2025.1547575 (PMC11831817; doi:10.3389/fpubh.2025.1547575)
Supplement: Supplementary file 1 [file Table_1.DOCX]

Supplementary Material

# Supplementary Table 1 The generalized least squares regression results on cognitive function, including the main and interaction effects (excluding IADL variable)

| **Variables** | **Categories** | **B** | **SE** | ***t*** | ***p*-value** | **Anova *p*** | **FDR q** |
| --- | --- | --- | --- | --- | --- | --- | --- |
| **Main effects** | | | | | | | |
| Intercept |  | 24.45 | 0.43 | 56.69 | <0.001 | - | - |
| Age, years (ref. ≥65 ~ ≤74) | ≥75 | -2.70 | 0.37 | -7.29 | <0.001 | - | - |
| Sex (ref. Men) | Women | -0.72 | 0.30 | -2.42 | 0.016 | - | - |
| Wave |  | -0.56 | 0.08 | -7.01 | <0.001 | - | - |
| Education level  (ref. Elementary school or less) | Middle school | 1.14 | 0.30 | 3.75 | <0.001 | <0.001 | <0.001  (<0.001) |
|  | High school | 1.69 | 0.27 | 6.35 | <0.001 |  |  |
|  | College or higher | 2.54 | 0.34 | 7.51 | <0.001 |  |  |
| Marital status (ref. Unmarried) | Married | 0.02 | 0.20 | 0.10 | 0.920 | - | 1  (0.595) |
| Drinking (ref. Non-drinker) | Former drinker | -0.58 | 0.19 | -3.02 | 0.003 | <0.001 | <0.001  (<0.001) |
|  | Current drinker | 0.27 | 0.19 | 1.43 | 0.152 |  |  |
| Regular exercise (ref. No) | Yes | 0.68 | 0.17 | 4.04 | <0.001 | - | <0.001  (<0.001) |
| BMI (kg/m^2^)  (ref. <18.5) | ≥18.5 ~ <23 | 0.23 | 0.26 | 0.88 | 0.380 | <0.001 | <0.001  (<0.001) |
|  | ≥23 ~ <25 | 0.71 | 0.27 | 2.58 | 0.010 |  |  |
|  | ≥25 ~ <30 | 0.78 | 0.29 | 2.72 | 0.007 |  |  |
|  | ≥30 | 0.59 | 0.52 | 1.13 | 0.258 |  |  |
| Cerebrovascular disease (ref. No) | Yes | -1.90 | 0.23 | -8.17 | <0.001 | - | <0.001  (<0.001) |
| CES-D-10 (range 0-30) |  | -0.11 | 0.02 | -5.55 | <0.001 | - | <0.001  (<0.001) |
| Social activity (ref. 0) | 1 | 0.75 | 0.16 | 4.62 | <0.001 | <0.001 | <0.001  (<0.001) |
|  | ≥2 | 0.88 | 0.22 | 3.91 | <0.001 |  |  |
| **Second order interaction effects** | | | | | | | |
| Age, years (≥75) × Sex (Women) | | -1.53 | 0.35 | -4.32 | <0.001 | - | <0.001  (<0.001) |
| Age, years (≥75) × Regular exercise (Yes) | | 0.35 | 0.28 | 1.24 | 0.216 | - | 0.825  (0.058) |
| Age, years (≥75) × Marital status (Married) | | 1.06 | 0.30 | 3.53 | <0.001 | - | 0.003  (<0.001) |
| Sex (Women) × Education level (Middle school) | | 0.57 | 0.41 | 1.40 | 0.162 | 0.021 | 0.110  (0.005) |
| Sex (Women) × Education level (High school) | | 0.91 | 0.40 | 2.26 | 0.024 |  |  |
| Sex (Women) × Education level (College or higher) | | 1.72 | 0.71 | 2.43 | 0.015 |  |  |
| Sex (Women) × CES-D-10 | | -0.04 | 0.02 | -2.29 | 0.022 | - | 0.110  (0.009) |
| Sex (Women) × Regular exercise (Yes) | | -0.40 | 0.22 | -1.79 | 0.073 | - | 0.313  (0.021) |
| Wave × CES-D-10 | | -0.03 | 0.01 | -3.84 | <0.001 | - | 0.001  (<0.001) |
| Wave × Social activity (1) | | 0.39 | 0.08 | 4.80 | <0.001 | <0.001 | <0.001  (<0.001) |
| Wave × Social activity (≥2) | | 0.50 | 0.12 | 4.24 | <0.001 |  |  |
| **Third order interaction effects** | | | | | | | |
| Age, years (≥75) × Sex (Women) × Regular exercise (Yes) | | 1.14 | 0.38 | 2.99 | 0.003 | - | 0.033  (0.003) |

B, Unstandardized coefficients; BMI, Body mass index; CES-D-10, The 10-item Center for Epidemiologic Studies of Depression Scale; FDR, False discovery rate; IADL, Instrumental activities of daily living; SE, Standard error.
