# Supplementary material for: Systematic identification and quantification of factors and their interactions with age, sex, and panel wave influencing cognitive function in Korean older adults
Source: Front Public Health. 2025 Feb 3;13:1547575. doi: 10.3389/fpubh.2025.1547575 (PMC11831817; doi:10.3389/fpubh.2025.1547575)
Supplement: Supplementary file 2 [file Table_2.DOCX]

Supplementary Material

# Supplementary Table 2 The delta R^2^ of the individual factors and the factor groups (excluding IADL variable)

| **Factors** | **Delta R^2^ (%) in the total population^a^** | | | | **Delta R^2^ (%) in each sub-population^a^** | | | | | | | |
| --- | --- | --- | --- | --- | --- | --- | --- | --- | --- | --- | --- | --- |
|  | **Individual**  **factors** | **Factor groups** | | | **Age (65~74y)** | | | | **Age (75y~)** | | | |
|  |  |  |  |  | **Men** | | **Women** | | **Men** | | **Women** | |
|  |  |  |  |  | **5^th^** | **8^th^** | **5^th^** | **8^th^** | **5^th^** | **8^th^** | **5^th^** | **8^th^** |
| Age | 6.6 | 7.8 | 19.6 | 7.8 | NA | | | | | | | |
| Sex | 1.2 |  |  |  |  |  |  |  |  |  |  |  |
| Wave | 0.7 |  |  |  |  |  |  |  |  |  |  |  |
| Education level | 3.6 | 5.1 |  | 14.4 | 3.0 | 2.2 | 0.3 | 2.8 | 2.8 | 1.8 | 4.5 | 2.7 |
| Marital status | 0.3 |  |  |  |  |  |  |  |  |  |  |  |
| Cerebrovascular disease | 1.0 |  |  |  |  |  |  |  |  |  |  |  |
| BMI | 0.3 | 7.8 | 7.8 |  | 12.2 | 18.1 | 9.8 | 17.3 | 12.5 | 19.8 | 14.3 | 22.7 |
| CES-D-10 | 3.6 |  |  |  |  |  |  |  |  |  |  |  |
| Drinking | 0.3 |  |  |  |  |  |  |  |  |  |  |  |
| Regular exercise | 1.0 |  |  |  |  |  |  |  |  |  |  |  |
| Social activity | 2.2 |  |  |  |  |  |  |  |  |  |  |  |
| Total | 31.9 | | | | 22.4 | 28.0 | 16.2 | 27.4 | 21.1 | 30.1 | 24.2 | 29.0 |

The factors were grouped according to the degrees of modifiability; The delta R^2^ for the factor groups were calculated from the results with the total population and sub-populations divided by the basic variables, respectively; ^a^The delta R^2^ was calculated with the difference between the model’s R^2^ with and without the specific factors or factor groups.

BMI, Body mass index; CES-D-10, The 10-item Center for Epidemiologic Studies of Depression Scale; IADL, Instrumental activities of daily living; NA, Not available.
